# How do people with MND and caregivers experience a digital mental health intervention? A qualitative study

**DOI:** 10.3389/fpsyt.2023.1083196

**Published:** 2023-02-02

**Authors:** Cathryn Pinto, Adam W. A. Geraghty, Francesco Pagnini, Lucy Yardley, Laura Dennison

**Affiliations:** ^1^School of Psychology, University of Southampton, Southampton, United Kingdom; ^2^Primary Care, Population Sciences and Medical Education, University of Southampton, Southampton, United Kingdom; ^3^Department of Psychology, Università Cattolica del Sacro Cuore, Milan, Italy; ^4^School of Psychological Science, University of Bristol, Bristol, United Kingdom

**Keywords:** motor neurone disease, psychological interventions, digital interventions, qualitative, wellbeing

## Abstract

**Objective:**

We urgently need to develop and evaluate more psychological interventions to support people with Motor Neurone Disease (MND) and caregivers. We used the person-based approach to develop a digital mental health intervention and conducted two studies to explore people’s experiences of using it.

**Methods:**

In Study 1, we conducted think-aloud interviews with 9 people with MND and 8 caregivers, and used findings to refine the intervention. In Study 2, 18 people with MND and 9 caregivers used the intervention for 6 weeks after which in-depth interviews were conducted. Data from both studies were combined and analysed using thematic analysis.

**Results:**

We developed 3 main themes around intervention acceptability, engagement, and usefulness. Participants highlighted the importance of accessibility and realistic presentation of information and support. Tailoring and timing intervention use to suit own needs, preferences, and disease stage was also important. Participants used the strategies presented to develop a positive outlook and regain some control. They also faced some challenges using these strategies in the context of dealing with progressive loss.

**Conclusion:**

People with MND and caregivers can find digital mental health interventions useful. Intervention accessibility and flexibility are important for developing acceptable and engaging interventions for MND.

## 1. Introduction

Motor Neurone Disease (MND) is a progressive, neurological disease which can lead to muscle weakness in the limbs, difficulties communicating, difficulties with swallowing and eating, breathlessness as well as other sensory symptoms and cognitive impairment (1). MND is a life-limiting disease with life expectancy typically 2–3 years from diagnosis (1). Currently there is no cure for MND, and treatment and care are centred around optimising quality of life. Some people with MND and their family members or caregivers can experience high levels of psychological distress and burden (2–5). Psychological support can help improve quality of life, however, research evidence for psychological interventions to support people with MND and caregivers is sparse, and there is an urgent need to develop and evaluate psychological interventions (6–9). Traditional face-to-face therapeutic interventions may not always be inclusive for people with MND with different physical symptoms, and they often require a large amount of time and commitment which can be a barrier for participation and engagement (10, 11). Digital mental health interventions (DMHIs) can provide an alternative format for psychological support and could be an accessible way to provide psychological support to people with MND and caregivers, particularly for those who have difficulties with mobility or speech, or for caregivers who need support that can fit around care tasks and schedules. In places such as the United Kingdom (UK) where demand for psychological support exceeds available resources, DMHIs can also be important for improving access to psychological resources (12, 13).

Research on DMHIs for people with MND and caregivers is in its infancy. So far, one online non-meditative mindfulness intervention for people with MND and caregivers, and another blended face-to-face and online acceptance and commitment therapy intervention for caregivers have been reported. Both studies were randomised controlled trials and showed promising results for improving wellbeing and quality of life, however, there were also some problems with adherence and drop out (14–16). Engagement with psychological interventions can be difficult with neurodegenerative conditions, and specifically with MND because of the variability of individual needs and symptoms, uncertainty about the disease and its progression, and challenges with acceptance and readiness to engage with interventions (17, 18). Therefore, understanding the experiences of people who might consider using DMHIs is important, and needed to develop engaging and acceptable psychological interventions for people with MND and caregivers.

We developed a DMHI that contained self-guided emotion regulation strategies, and practical tips to deal with distress and improve wellbeing. The aim of the current paper was to explore how people with MND and caregivers engaged with and used the intervention we developed which would help us refine and optimise it. We also wanted to develop a broader understanding of how people with MND and caregivers engage with and use self-guided DMHIs.

## 2. Materials and methods

### 2.1. Design

**FIGURE 1:**
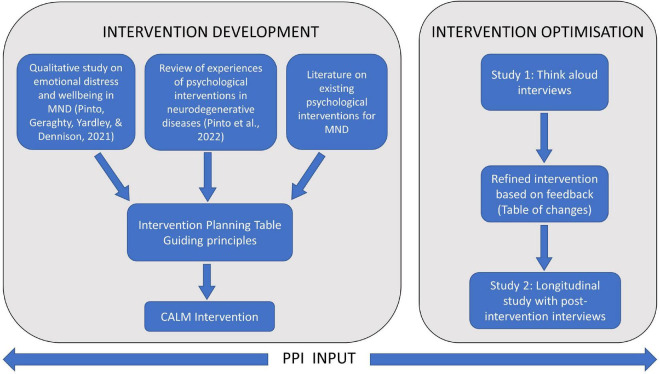
Process of intervention development and optimisation.

We used the person-based approach to develop and optimise the intervention. This approach largely uses qualitative, iterative methods, and incorporates in-depth feedback from target users at different stages of development, optimisation, and evaluation (19, 20). The aim of this approach is to understand the target users’ context, their views on the intervention, and use this data to design interventions that are persuasive, feasible, and relevant to users (21). Our overall intervention development and optimisation process is shown in Figure 1. The two studies presented in this paper were nested within the intervention optimisation stage. Both studies were designed to seek user feedback in order to refine and optimise the intervention prior to more rigorous evaluation. In study 1, we conducted think-aloud interviews with people with MND and caregivers to examine the acceptability of the intervention content and presentation, and findings were used to refine the intervention. In study 2, different people with MND and caregivers used the intervention for 6 weeks and were subsequently interviewed about their experiences.

### 2.2. Intervention

**FIGURE 2:**
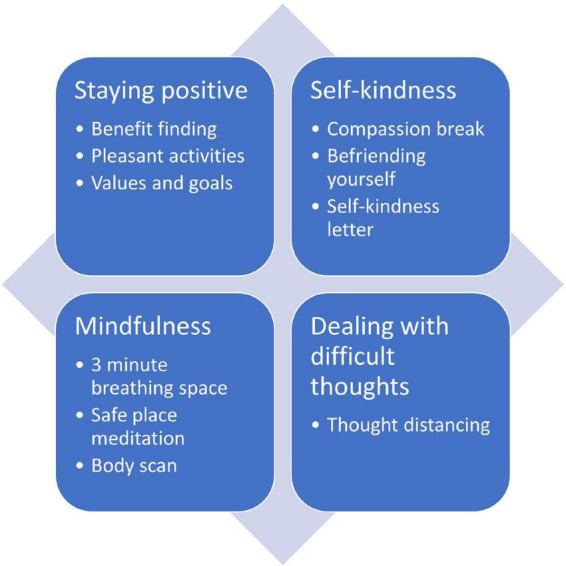
Self-guided emotion regulation strategies used in the intervention.

**FIGURE 3:**
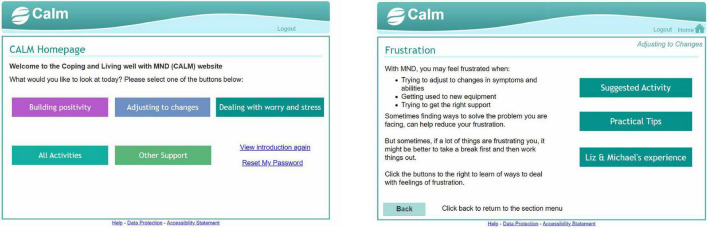
Example 1 of pages from the CALM website.

**FIGURE 4:**
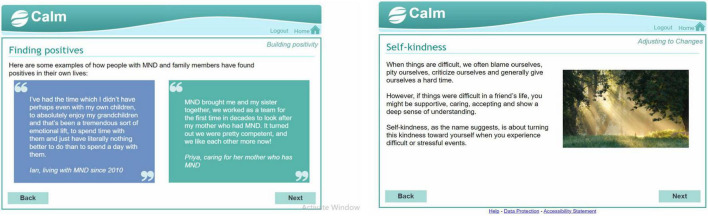
Example 2 of pages from the CALM website.

**FIGURE 5:**
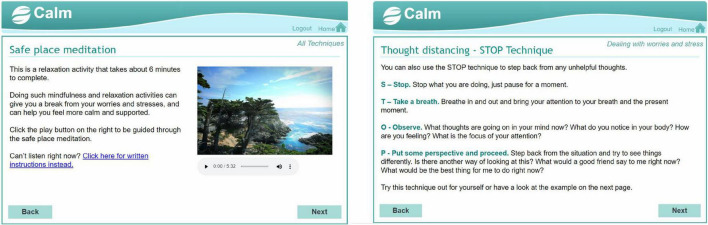
Example 3 of pages from the CALM website.

We created a digital mental health intervention in the form of an interactive website called Coping and Living well with MND (CALM). The intervention planning was guided by a combination of theory (22), previous research on effective psychological interventions for MND [e.g., (23, 24)], and qualitative research on coping strategies, preferences for and engagement with psychological interventions (17, 25). See Supplementary materials 1, 2 for the intervention planning table and guiding principles. The website contained activities from cognitive behavioural therapy, mindfulness, acceptance, and commitment therapy, and compassion-focussed therapy (see Figure 2). The website was meant to be used independently by people with MND and caregivers without any professional facilitation or support. It did not have a guided format and allowed people to choose relevant sections or activities. The information and activities were adapted to be short and accessible to people with various levels of physical ability. The layout and navigation were designed to be simple and easy to use for people who may have different physical and cognitive difficulties (e.g., large font and buttons, limited information per page, compatible to use with screen readers). The design was based closely on an intervention for managing distress in primary care settings (26). Examples or quotes from people with MND and caregivers were used throughout the website to demonstrate how the information and activities were relevant to MND. See Figures 3–5 for examples of pages from the website.

### 2.3. Ethics

We obtained ethical approval from the University of Southampton ethics committee (ERGOII-61216).

### 2.4. Participants and recruitment

Participants were eligible if they had a diagnosis of MND or were a caregiver for someone with MND and had mental capacity to give informed consent. They needed to be able to participate in either a video call or written feedback and have access to an electronic device and the internet to complete online surveys and use the CALM website. Recruitment was carried out through the UK Motor Neurone Disease Association (MNDA), a charity that supports people with MND. Information about the research was placed in their newsletters, website, and social media platforms. A call for participants for study 1 was put out first, and another call for study 2 was put out when study 1 was complete. Interested individuals contacted the researcher to discuss participation, were given an information sheet and filled a consent form to take part. Different participants took part in studies 1 and 2.

For study 1, we aimed to recruit up to 10 people with MND and 10 caregivers, and recruitment stopped when data saturation was reached (i.e., no new intervention changes were suggested). For study 2, we aimed to recruit 15–20 people with MND and 10–15 caregivers based on the following criteria. For people with MND, we purposively selected people with a range of symptoms (difficulties using arms, legs, and with speech) and varying lengths of time since diagnosis (early and later stages of the disease). We recruited caregiver participants who were currently caring for someone with MND and wished to use the intervention for their own emotional needs.

### 2.5. Data collection

Data collection took place between October 2020 and July 2021, and was conducted remotely due to the COVID-19 pandemic. The researcher discussed with participants the most appropriate and comfortable way of conducting the interviews (e.g., *via* phone, video call, or written responses). Where requested or necessary due to physical impairments, participants were interviewed as a patient-caregiver dyad.

#### 2.5.1. Study 1

Participants completed a brief online questionnaire that captured demographic and clinical information which was initially used to guide purposive sampling and subsequently used to report characteristics of the sample. This questionnaire included two validated scales. Participants with MND completed the Amyotrophic Lateral Sclerosis Assessment Questionnaire (ALSAQ-40), a self-report of health status capturing physical mobility, activities of daily living, eating and drinking, communication, and emotional reactions (27). Caregivers completed the Zarit burden interview (ZBI-12) (28). Participants were also asked to self-report any cognitive difficulties and caregivers reported if their family member experienced any cognitive difficulties. Interviews were conducted at a date and time convenient to participants *via* video call or phone. All interviews were conducted by a researcher (CP), who had training and experience in qualitative methods and had worked clinically with people with neurodegenerative diseases. During the interview, participants viewed the CALM website on their own devices and simultaneously answered interview questions. Think-aloud interviews followed a semi-structured format with a set of standard questions, and follow-up questions based on the particular webpages participants looked at and their initial reactions (see Supplementary material 3). Feedback was logged in a “table of changes,” discussed regularly with the research team and used to refine the intervention (see Supplementary material 4).

#### 2.5.2. Study 2

As with Study 1, participants completed a brief questionnaire online capturing demographic and clinical details (ALSAQ-40 and ZBI-12). Participants were sent a link to the website with brief instructions about logging in and using the website, and a researcher checked in with them after 2 weeks. After 6 weeks, the researcher contacted participants to arrange the video call or phone interviews. Participants who had difficulties with speech were sent a questionnaire with open-ended questions so they could type their feedback; follow-up questions were emailed if responses needed more elaboration. Interviews were semi-structured and covered questions about participants’ overall thoughts and feelings about the intervention, with more detailed questions about using specific advice or strategies (see Supplementary material 5 for the interview topic guide).

### 2.6. Patient and public involvement

Patient and public involvement (PPI) members (2 people with MND and 2 former caregivers) had input throughout the entire process of intervention development. They were recruited through the MNDA charity, and expressions of interest following dissemination activities and participation in previous studies for the same project. Initial versions of the intervention were shared with PPI members and feedback was used to improve presentation and navigation. Data collection procedures were reviewed by PPI members, especially the open-ended questionnaire for people who had difficulties with speech, and feedback was used to make the process smoother and interview questions clearer. Some PPI members also gave their input on the preliminary findings.

### 2.7. Data analysis

All interviews were transcribed verbatim and identifying characteristics were removed. Data from the think-aloud interviews in study 1 was logged in the “table of changes” and used by the research team to refine the intervention (see Supplementary material 4). This included specific feedback about the presentation and phrasing of information on the webpages. Interview data from study 2 was used to understand participants’ experiences of using the intervention. Data from the think-aloud interviews where participants expressed their views and perceptions about using and engaging with the intervention was added to the data set for analysis. Patient and caregiver interviews were coded separately with the intention of exploring differences in views and experiences and then compared at the theme development stage. As there were very minor differences in the emerging codes, themes were developed drawing on data from both patient and caregiver codes.

We used an inductive thematic analysis to draw themes in relation to experiences of engaging with and using DMHIs and emotion regulation strategies in MND (29). After familiarisation with the interviews through repeated reading of transcripts, CP began coding the data inductively. A list of codes was developed and reviewed within the research team to identify interesting aspects and patterns of the data. At this stage, diagrams were used to map out the relationship between different codes across the dataset and identify candidate themes relevant to the research question. Descriptions of each candidate theme were written out and codes were revisited to make sure the descriptions reflected the data. The themes were reviewed by the research team and changes were made to the themes and subthemes. This process involved adding subthemes that were originally considered very descriptive, but were re-worked and included as they formed an important part of the narrative of participants’ experience. Themes were defined and theme names were refined iteratively through discussions within the research team and through engaging with the wider literature. Presenting preliminary findings through project dissemination activities also helped further refine the essence of each theme and ensure the theme names reflected this. The findings were written for publication, together with appropriate data extracts that helped convey the story of how participants engaged with the intervention and used the advice and strategies.

## 3. Results

For study 1, 14 think-aloud interviews were conducted with 9 people with MND and 8 caregivers. From these interviews, 4 interviews were joint interviews with patient-caregiver dyads, and the rest were conducted individually. A total of 13 interviews were conducted by video call and 1 by telephone.

For study 2, 34 participants consented to the study. From these, 7 participants dropped out of the study (6 participants dropped out before looking at the intervention and 1 participant dropped out after briefly accessing the intervention). The remaining participants (18 people with MND and 9 caregivers) accessed the intervention and took part in post-intervention interviews. A total of 26 post-intervention interviews were conducted, only one was conducted as a joint interview with patient and caregiver, the rest were conducted individually. A total of 18 interviews were conducted *via* video call, 1 *via* phone, and 7 *via* written feedback.

A total of 27 people with MND and 17 caregivers participated across both studies. Demographic and clinical details of the total sample can be found in Table 1.

**TABLE 1:** Demographic and clinical details of the sample.

Characteristic	People with MND (*n* = 27)	Caregivers (*n* = 17)
**Age**		
Mean (range)	63.96 (48–92)	54.47 (20–73)
**Gender**		
Man	14	5
Woman	13	12
**Ethnicity**		
White British/Irish/Other	27	16
Asian/Asian British	0	1
**Relationship to person with MND**		
Spouse/partner		11
Son/daughter		5
Sibling		1
**Education**		
Up to GCSE or equivalent	7	1
A levels or equivalent	5	2
Graduate level	9	10
Postgraduate level	6	4
**Diagnosis**		
ALS	17	
Primary lateral sclerosis	3	
Progressive bulbar palsy	3	
Progressive muscular atrophy	2	
Kennedy’s disease	2	
**Time since diagnosis**		
Less than 1 year	9	6
1–5 years	8	6
More than 5 years	10	5
Health status (ALSAQ-40)* Median (IQR)		
Physical mobility	45 (28.75)	
Activities of daily living/independence	50 (28.75)	
Eating and drinking	16.67 (45.83)	
Communication	14.29 (71.43)	
Emotional functioning	40 (27.50)	
**Caregiver burden (Zarit burden interview)**		
No to mild burden (0–10)		1
Mild to moderate burden (10–20)		5
High burden (>20)		11
Cognitive Impairment (self-reported)	5	6

*ALSAQ 40: Scores on each subscale range from 0 to 100 with a higher score representing a greater degree of impairment.

**FIGURE 6:**
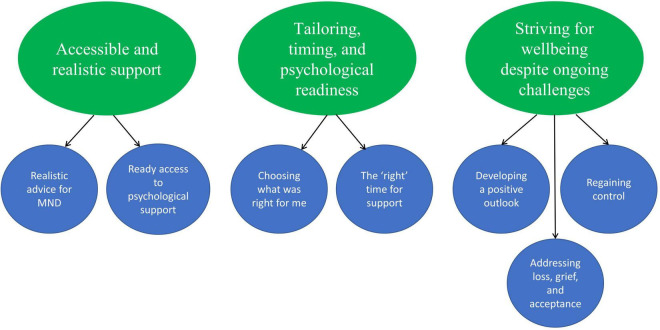
Thematic map of main themes and corresponding subthemes.

We developed 3 main themes and 7 subthemes which reflect key aspects of engaging with and using the intervention and the specific emotion regulation strategies presented (see Figure 6).

### 3.1. Accessible and realistic support

This theme and respective subthemes highlight the importance and meaning of accessibility of support for people with MND and caregivers. It includes a discussion of the accessibility and realistic presentation of specific advice and strategies, and of having ready access to psychological support.

#### 3.1.1. Realistic advice for MND

It was important for people with MND and caregivers that the information and activities were presented in a realistic way that matched their experience of living with MND and the realities of MND care. The information and strategies needed to be presented in a way that was sensitive to the symptoms and needs of people with MND and caregivers.

*It didn’t tell you just to sort of pick yourself up and go for a walk, up and down the stairs or something when you might not well be able to do that, you’d love to do that but you can’t do that, and so it was a bit more appropriate I think to people with motor neuron (Rachel, person with MND, 55 years, 2 years since diagnosis)*.

Participants stressed the importance of framing advice and strategies in an accessible way particularly for people with very limited physical ability or for those who required assistance or practical support to be able to follow some of the advice on the website. This was discussed with particular reference to sections that encouraged people to do more activities that were meaningful and brought them joy.

*It may be helpful to say when you are planning your list, it may be helpful to discuss with your friend or family member so they can help with any preparations or adaptations (Priya, caregiver, 40 years, 3 years since diagnosis)*.

Participants also felt that some advice could be phrased more realistically and acknowledge that it may sometimes be difficult to follow because of the realities of living with or caring for someone with MND. One example of this was the thought distancing or mindfulness activities that may provide temporary relief, but with MND worries often crop up again. Participants felt this could be phrased in a way where people could anticipate the benefits of the activity but were also aware of the effort it may take. Similarly, for caregivers it may be difficult to take time out to look after themselves and take a break from caring responsibilities. Some described a tendency to neglect taking care of themselves in order to take care of the person with MND, and feeling guilty when they did take a break.

*My mum looks after my dad and is a full-time carer and it’s fine saying take some time for yourself, but if my dad has to go to the toilet, then she has to help with that, you’re always on call (Tina, caregiver, 48 years, 6 months since diagnosis)*.

*I was struggling to find time or be able to actually leave the house to go out to an exercise class, to travel to it, to do the class, to travel back because of the time it takes (Rose, caregiver, 69 years, 1.5 years since diagnosis)*.

#### 3.1.2. Ready access to psychological support

Having a website that provides timely and continuous access to some psychological support was seen as important. Finding and accessing psychological support for MND can be difficult, and some participants described their struggle with the lack of psychological support available. They highlighted the value of being able to readily access tips and strategies and being introduced to different kinds of things that might be helpful. The availability of psychological support through the CALM website was reassuring for some participants who took comfort in knowing that support was there should they ever need it.

*It’s very helpful knowing that this stuff is all there, for when you get in such a state that you can’t think for yourself really (Elaine, caregiver, 73 years, 6 years since diagnosis)*.

Having psychological support or advice that was readily available was seen as helpful, especially as things change with the disease. One participant described the time-bound nature of other forms of psychological support, and how having continuous access to some psychological support through a website was important.

*I’ve done the mindfulness course, I’ve done this CBT, I’ve seen the counsellors and then it all stops. Do you know what I mean? So having something like that… OK, I recognize a lot of the little practical tips and the ideas, but I haven’t got access to them without this site so that it’s helped in that respect. And anytime you do any of those things, even if it’s just a quick body scan or a 3-min breathing exercise or something, it has a positive effect (Rachel, person with MND, 55 years, 2 years since diagnosis)*.

### 3.2. Tailoring, timing, and psychological readiness

In this theme, we present participants’ views about tailoring their use of the website to their own needs and preferences. Having the choice to decide when and how to engage with support from the website was important for people with MND and caregivers, and the diversity of views are discussed in the subthemes.

#### 3.2.1. Choosing what was right for me

Participants valued being able to choose sections or activities from the website that were relevant to them. It was important for them to choose activities that were in line with their personality or coping style. For example, participants who were familiar with yoga and or cognitive behavioural therapy techniques were drawn to activities like mindfulness or goal setting, respectively. Others described how they aimed to stay positive and keep busy as a way of coping with MND, and were not tempted to try activities like mindfulness or thought distancing that required introspection about thoughts and feelings.

*I did look through every section at first, but I knew what would be for me and what wouldn’t and the distancing [thought distancing]… I listened to it and went away, but I thought “no, this isn’t for me, so that’s fine” (Liz, person with MND, 56 years, 9 years since diagnosis)*.

How participants coped and what was important to them changed based on what stage they were in the disease. Therefore, having a range of strategies to choose from allowed people to select ones that were appropriate to them at that time. Conversely, participants also decided not to use strategies at certain times. For example, one participant described not wanting to try the mindfulness activities while he still had physical ability to actively do things that would help him feel better. Similarly, one carer explained that she was not at the stage where she was ready to look at the “building positivity” section. For people with MND, the body scan mindfulness activity was a particular activity whose perceived applicability and value appeared to be strongly influenced by disease stage.

*Practical tips and getting rid of the negative thoughts and things like that, that’s important to me at the moment, so that’s where I went to, just depends on how I felt when I was looking at the website (Rachel, person with MND, 55 years, 2 years since diagnosis)*.

*No, I did not try these activities [in the building positivity section]. That is not to say they are not necessarily helpful activities, I think I am not ready for these just yet. One stage at a time (Carmen, caregiver, 57 years, 5 months since diagnosis)*.

Some participants described how they preferred to learn and use the strategies from the website in their spare time, whereas others preferred using them when they were struggling with particular emotions. Not having a fixed format for the website made it easier for participants to adapt website use to these preferences. Both caregiver and patient participants suggested including more activities on the website because having options and choice was important with MND.

#### 3.2.2. The “right” time for support

Participants felt it was important to make their own decisions about the best time to engage with psychological support from the CALM website. This was a subjective decision based on participants’ own assessments of how they were coping, and participants at similar stages could have very diverse views. For some participants at an early stage of the disease, their symptoms did not have much of an effect on their daily life and they explained that the website was currently not as useful for them. However, they could see that it would be useful later on, when they were more affected by symptoms or had more caring responsibilities.

*To be quite honest because of my condition at the moment, although I’m slowly losing the ability in my leg, I’m sort of pretty positive about it all… An awful lot of it didn’t really refer to how I feel if you know what I mean? I’m sort of still relatively happy and positive. I can imagine once things get worse, I might find it of more interest really (Michael, person with MND, 79 years, 2 years since diagnosis)*.

There were some participants, however, who would have preferred to receive support from a website like this early on, closer to when they were diagnosed. These participants wanted support to deal with the shock of diagnosis, uncertainty about what was going to happen, and the emotional impact of receiving the diagnosis. They explained their journey finding appropriate psychological support or developing their own coping strategies, and said that the advice and strategies from the website mirrored what they had learned or now do. The website served as validation or confirmation that they were on the right track, and was a useful reminder and motivator to continue to use these strategies. A few participants wanted to be prepared for the future, and the website prompted them to think of activities they could do to look after their mental health when their symptoms got worse.

*It’s helped me because it has made me do those things again or allowed me to do those things, and reminded me of the positive effect of some of those exercises and ways of thinking and it just sort of, just confirms it really (Rachel, person with MND, 55 years, 2 years since diagnosis)*.

Some participants felt the website came at the right time for them. They were either experiencing new symptoms or struggling to adjust to new equipment, and felt that the information and strategies could be applied to the problems they were facing.

*I particularly liked this section [self-kindness activity]. I found it helped me through a tough time I was having recently when I was introduced to RIG [radiologically inserted gastronomy] tube feeding and experienced adverse side effects. This section and activities allowed me to look at my thinking “I feel I’m not a strong person to cope with this disease, other people cope” (Jane, person with MND, 61 years, 1.5 years since diagnosis)*.

### 3.3. Striving for wellbeing despite ongoing challenges

This theme captures how people with MND and caregivers used the strategies in the website to cope with emotional difficulties and improve wellbeing. It also sheds light on the struggles with using some of these strategies to maintain wellbeing when facing progressive loss.

#### 3.3.1. Developing a positive outlook

Participants used advice and strategies from the website to develop and maintain a more positive way of coping with MND. Some participants looked at activities they were currently doing, and found it helpful to realise that they were in line with their values. Others planned to do more activities that would bring them joy which allowed them to look towards the future in a more positive way.

*I think I’m someone that likes to look ahead, you know, so having some new goals and new values is very useful, it’s a positive thing to focus on (Jo, person with MND, 57 years, 10 months since diagnosis)*.

People with MND often gave examples of how they made adaptations to continue doing activities they enjoyed, or how they planned to do this when symptoms got worse. Caregivers also described how they adapted activities they enjoy to fit around their caring tasks.

*I like gardening and my husband will sit me down outside and bring everything to me so that I can still plant up planters and hanging baskets but everything is brought to me (Liz, person with MND, 56 years, 9 years since diagnosis)*.

For other participants, exploring different ways of thinking about their situation gave them a different and more positive perspective. This different perspective came from either following the website activities or from reading examples or quotes from people with MND and caregivers explaining how they coped with difficulties.

*Just reminding yourself that there are other ways of thinking of things, and that sticks in my mind a lot there. Just stopping, taking up a breath, just taking a bit of time to say, “OK, that’s what I’m thinking, that life isn’t worth or whatever” you know… But there are other ways of thinking about things and you need to just… “what are they,” there must be some other way of looking at it. And then just sitting and trying to think that through, that helped quite a bit (Rachel, person with MND, 55 years, 2 years since diagnosis)*.

Some people with MND said they developed a more positive outlook through looking at themselves and their body not as failing, but in a positive and kinder way.

*From the motor neurone disease point of view, it’s accepting that your body is failing and not getting too angry with that part of your body and being accepting of it, being kind to it… So yeah, I thought that [body scan activity] was good (Jo, person with MND, 57 years, 10 months since diagnosis)*.

The activities in the building positivity section were seen as relevant and useful, and in keeping with some people’s coping strategies. The activities prompted some to recognise and list positive things in their life. However, some people found that these activities were difficult to do on certain days, when they were stressed or in times of crisis. In particular, people with MND who had very limited ability or no family or social support found it difficult to follow some of the advice or strategies in the building positivity section. Caregivers who witnessed rapid decline in the health of their family member also struggled with some of the activities to promote positivity.

*Positive thoughts are easy when you can do things for yourself, but hard when you can’t (Chris, person with MND, 67 years, 9 months since diagnosis)*.

*It all makes sense really, to try and find the positives in what’s happening. I did read that several times because obviously there are times when you think everything is negative and so I have been going on that and thinking about ways that I could improve my positivity (Diana, person with MND, 61 years, 5 months since diagnosis)*.

#### 3.3.2. Regaining control

Participants used advice and activities from the website to feel more in control over what was happening. Both caregivers and people with MND spoke about struggling with thoughts about the future, triggered particularly after a setback or symptoms getting worse. In these situations, using some of the advice and strategies from the website (e.g., mindfulness and thought distancing activities) helped them calm down and step back from the situation that was overwhelming them. This then helped them approach the problem in a different and often more constructive way.

*Fear and anxiety took over as I’ve been so independent regarding my daily hygiene and to allow a stranger into my home to help me filled me with dread instead of ease and peace of mind. With CALM [website], [STOP technique for thought distancing] I was able to take a look at and acknowledge this worry and fear, see that these new changes will benefit and make things safer and help me. Surely I want the best for myself. Stopping and observing allows a little clarity in the situation and makes for a good outcome (Jane, person with MND, 61 years, 1.5 years since diagnosis)*.

*I do find those [mindfulness activities] really useful cause it means you can just focus on something else rather than just powering forward and doing everything else. I do find it really useful to have that time to just stop and think (Maria, caregiver, 22 years, 8 months since diagnosis).*

Participants also found it helpful to focus on the here and now, particularly when there were worrying thoughts about the future. The strategies helped them focus on things they can do, as opposed to things they could no longer do. However, some participants explained how it was difficult to focus on the present whilst also needing to plan for the future.

*We really do, you have to think ahead of ourselves because [name of person with MND]’s not going to get better. So we have to put things in situ that will help. So it’s very…it’s odd cause you are in the mindfulness zone and then all of a sudden you think “Gosh, I really do have to think of this” (Stacy, caregiver, 68 years, 1.5 years since diagnosis).*

Participants felt that the website strategies gave them something positive or constructive to focus on, which gave them more control over their situation. Having access to the information and strategies from the website increased their confidence in their ability to deal with difficult emotions.

*I’ve done the activities when I’ve been feeling down or been in a quiet and reflective mood, they help me focus on positive things that I can do to help me feel as though I’m taking some control over what is happening to me (Peter, person with MND, 54 years, 6 months since diagnosis)*.

*I’m more positive I guess, and know I have ways of, you know, when I do have bad days say I’ll go and listen to the body scan or any of the audios, because I can just close my eyes and listen and just refocus (Diana, person with MND, 61 years, 5 months since diagnosis)*.

#### 3.3.3. Addressing loss, grief, and acceptance

Participants described a need to address and deal with grief and loss, whilst they strived to be positive and engage with strategies to improve their wellbeing. There were constant reminders of things they could no longer do or things that alluded to future loss. These triggers sometimes came from features in the website (e.g., pictures of nature or people, lists of activities, quotes from others). Reminders of loss also were also present when adapting activities or using equipment to continue to do enjoyable and meaningful activities.

*The sadness didn’t leave me it stayed there, it was just temporarily overtaken by my enjoyment of either seeing close family and friends, going somewhere or doing another activity. Even though I was doing things I enjoyed my MND was still impacting on the activity (Peter, person with MND, 54 years, 6 months since diagnosis)*.

Participants wanted more of an acknowledgement of the grief and loss they were experiencing. Accepting loss was seen as necessary in order to be able to do some of the activities. Some participants felt they needed more guidance on dealing with this grief and loss.

*I would like an acknowledgement earlier on in this, that the pleasant things have changed massively. So if I had been doing this pre MND obviously some of these things may be on my list, but there’s many, many things that wouldn’t be on that list, that can’t be on that list. So there’s a big sense of loss for me when I think about pleasant activities (Wendy, person with MND, 54 years, 6 months since diagnosis)*.

Acceptance was also important in order to be open to receiving the information in the website. Participants described this initial struggle with acceptance, but also explained how they experienced benefit when they persisted with the advice and strategies. In some cases, the information on the website facilitated this acceptance by giving people a chance to think about or assess how they were feeling or coping, and giving people permission to think about and address their emotions.

*I think the very first time I went into the website it made me cry and I think that’s because it was a real acknowledgement of what I was going through. Maybe that’s the first time, I’ve really, kind of sat down and gone “gosh, this is huge.” That was quite powerful, but I’ve gotten over that as I started using it [the website] but it did have quite a powerful effect the first time I used it (Jo, person with MND, 57 years, 10 months since diagnosis)*.

## 4. Discussion

In summary, people with MND and caregivers found the intervention useful for developing a more positive outlook and regaining some control over their lives. However, participants also needed to acknowledge and deal with losses and accompanying grief whilst they attempted to use these strategies for their wellbeing. We found that accessibility and realistic presentation of information and support were important for people with MND and caregivers. It was also important for participants to decide what was right for them, based on individual needs and preferences as well as what stage of the disease they were at. Our findings relate to the CALM website, however, the themes are applicable more generally to psychological support for MND and using self-guided DMHIs with similar populations.

Similar to our findings, other studies have reported an increase in acceptance and perceived control from using psychological interventions (16, 30). People with MND and caregivers also exert control by choosing to engage with services, choosing when to accept help from others or to use assistive devices (25, 31, 32). We found that there was considerable variability between participants about which strategies were right for them and the right time for support from a psychological intervention. Psychological support needs to be flexible and offered at different points in the disease trajectory as symptoms and needs change (33, 34). DMHIs can offer this flexibility, however, variability in use is to be expected.

Emotion regulation strategies may also need to be adapted or repeated to help people stay positive and in control in the context of experiencing loss. Experiences of loss and positivity are intertwined and both can be part of the process of adaptive coping. The dual process model of coping with bereavement explains that coming to terms with loss involves oscillating between confronting the loss and processing it, and avoiding the loss or attending to practical consequences of the loss (35). Therefore, wellbeing in progressive conditions like MND needs to be seen in terms of adaptability and flexibility, where people shift to different ways of coping over time and with the right support. Approaches such as Acceptance and Commitment Therapy can be helpful as they enable acceptance of both positive and negative experiences in the context of personal values, and the resulting psychological flexibility acts as a buffer to distress (36).

Engagement with DMHIs is facilitated when interventions can be integrated into people’s daily routines (37). With MND, additional practical support (e.g., respite for caregivers or assistive devices for people with MND) may be required to enable people to use the self-guided emotion regulation strategies. Although DMHIs can improve access to psychological support, they also have some limitations. They can bring up difficult emotions that may need professional support, or they may not be an appropriate tool to use at certain points (e.g., in crisis or very stressful times). Providing choice about support in online and face-to-face formats can help overcome some of these challenges (38). Self-guided support may lead to people choosing strategies they are comfortable with and not trying out new ways of coping. In these instances, a therapist-supported or hybrid or blended approach to DMHIs may be valuable, where individuals can be guided through helpful strategies and where strategies and feedback can be personalised (16).

### 4.1. Strengths and limitations

The user-centred approach to intervention development and optimisation was a strength and enabled us to anticipate some of the issues with engagement and acceptability. Additionally, we obtained views of participants with a range of symptoms, participants at different stages in the disease, and those who looked at the intervention and decided not to use it. This gave us a broader understanding of the limits and challenges of using DMHIs with MND.

Intervention development and the studies conducted to optimise the intervention were conducted by the same researcher and this may have influenced the findings. The researcher took steps to be reflexive about researcher influence through careful framing of interview questions, asking participants for both positive and negative feedback on the intervention, using field notes to reflect on data collection processes and interviewees’ responses, discussing feedback from the interviews with the research team and making changes to the intervention collaboratively, regularly discussing codes and developing themes with the research team. The 6-week period may not have been sufficient for some participants to learn and apply the strategies to problems they encountered. The one-off interviews at the end of the 6-week period were subject to participants’ recall and may not have provided a complete picture of intervention engagement. Another limitation is that self-motivated people who were interested in improving their mental health or people comfortable with using digital support may have come forward to participate in the study. Recruiting participants from the national health service could have helped access the views of a broader range of participants especially those who may be reluctant to seek digital mental health support.

## 5. Conclusion

People with MND and caregivers can find DMHIs useful, particularly for developing a positive outlook and regaining control. It is important to pay attention to accessibility, choice, and flexibility when designing DMHIs for MND so people can tailor their use to meet their own needs. DMHIs can help improve access to psychological support for people with MND and caregivers. However, self-guided psychological support has its limitations and should be provided as an option alongside other forms of psychological support.

## Data availability statement

The raw data supporting the conclusions of this article will be made available by the authors, without undue reservation.

## Ethics statement

The studies involving human participants were reviewed and approved by University of Southampton ethics committee (ERGOII-61216). The patients/participants provided their written informed consent to participate in this study.

## Author contributions

CP main contributor to the design of the study, ethics application, recruitment, data collection and analysis, reporting, and publication of findings. AG and LD involved in obtaining funding for the study, contributed to the study design, supervised CP during recruitment, data collection and analysis, contributed to the reporting, and publication of findings. FP contributed to discussions and provided support that led to the intervention development, provided feedback on the reporting, and publication of findings. LY involved in obtaining funding for the study, provided feedback on the reporting, and publication of findings. All authors contributed to the article and approved the submitted version.

## References

[1] Moore C McDermott C Shaw P. Clinical aspects of motor neurone disease. *Medicine.* (2008) 36:640–5. 10.1016/j.mpmed.2008.09.001

[2] Averill A Kasarskis E Segerstrom S. Psychological health in patients with amyotrophic lateral sclerosis. *Amyotroph Lateral Scler.* (2007) 8:243–54. 10.1080/17482960701374643 17653923

[3] Burke T Galvin M Pinto-Grau M Lonergan K Madden C Mays I Caregivers of patients with amyotrophic lateral sclerosis: investigating quality of life, caregiver burden, service engagement, and patient survival. *J Neurol.* (2017) 264:898–904. 10.1007/s00415-017-8448-5 28280986

[4] Chen D Guo X Zheng Z Wei Q Song W Cao B Depression and anxiety in amyotrophic lateral sclerosis: correlations between the distress of patients and caregivers. *Muscle Nerve.* (2015) 51:353–7. 10.1002/mus.24325 24976369

[5] Pagnini F. Psychological wellbeing and quality of life in amyotrophic lateral sclerosis: a review. *Int J Psychol.* (2013) 48:194–205. 10.1080/00207594.2012.691977 22731673

[6] Aoun S Bentley B Funk L Toye C Grande G Stajduhar KJ. A 10-year literature review of family caregiving for motor neurone disease: moving from caregiver burden studies to palliative care interventions. *Palliat Med.* (2013) 27:437–46. 10.1177/0269216312455729 22907948

[7] Burke T O’Raghallaigh J Maguire S Galvin M Heverin M Hardiman O Group interventions for amyotrophic lateral sclerosis caregivers in Ireland: a randomised controlled trial protocol. *BMJ Open.* (2019) 9:e030684. 10.1136/bmjopen-2019-030684 31542756 PMC6756338

[8] Gould R Coulson M Brown R Goldstein L Al-Chalabi A Howard R. Psychotherapy and pharmacotherapy interventions to reduce distress or improve well-being in people with amyotrophic lateral sclerosis: a systematic review. *Amyotroph Lateral Scler Frontotemporal Degener.* (2015) 16:293–302. 10.3109/21678421.2015.1062515 26174444

[9] Zarotti N Mayberry E Ovaska-Stafford N Eccles F Simpson J. Psychological interventions for people with motor neuron disease: a scoping review. *Amyotroph Lateral Scler Frontotemporal Degener.* (2021) 22:1–11. 10.1080/21678421.2020.1788094 32657152

[10] Marconi A Gragnano G Lunetta C Gatto R Fabiani V Tagliaferri A The experience of meditation for people with amyotrophic lateral sclerosis and their caregivers–a qualitative analysis. *Psychol Health Med.* (2016) 21:762–8. 10.1080/13548506.2015.1115110 26584831

[11] Van Groenestijn A Schröder C Visser-Meily J Reenen E Veldink J Van Den Berg L. Cognitive behavioural therapy and quality of life in psychologically distressed patients with amyotrophic lateral sclerosis and their caregivers: results of a prematurely stopped randomized controlled trial. *Amyotroph Lateral Scler Frontotemporal Degener.* (2015) 16:309–15. 10.3109/21678421.2015.1038276 26087303

[12] Davies S. *Annual report of the chief medical officer: public mental health priorities: investing in the evidence.* London: Department of Health and Social Care (2014).

[13] Hollis C Sampson S Simons L Davies E Churchill R Betton V Identifying research priorities for digital technology in mental health care: results of the James Lind alliance priority setting partnership. *Lancet Psychiatry.* (2018) 5:845–54. 10.1016/S2215-0366(18)30296-7 30170964

[14] Pagnini F Phillips D Haulman A Bankert M Simmons Z Langer E. An online non-meditative mindfulness intervention for people with ALS and their caregivers: a randomized controlled trial. *Amyotroph Lateral Scler Frontotemporal Degener.* (2022) 23:116–27. 10.1080/21678421.2021.1928707 34027769

[15] De Wit J Beelen A Drossaert C Kolijn R Van Den Berg L SchrÖder C Blended psychosocial support for partners of patients with ALS and PMA: results of a randomized controlled trial. *Amyotroph Lateral Scler Frontotemporal Degener.* (2020) 21:344–54. 10.1080/21678421.2020.1757114 32362155

[16] de Wit J Vervoort S van Eerden E van den Berg L Visser-Meily J Beelen A User perspectives on a psychosocial blended support program for partners of patients with amyotrophic lateral sclerosis and progressive muscular atrophy: a qualitative study. *BMC Psychol.* (2019) 7:35. 10.1186/s40359-019-0308-x 31202270 PMC6570885

[17] Weeks K Gould R Mcdermott C Lynch J Goldstein L Graham C Needs and preferences for psychological interventions of people with motor neuron disease. *Amyotroph Lateral Scler Frontotemporal Degener.* (2019) 20:521–31. 10.1080/21678421.2019.1621344 31298054

[18] Pinto C Geraghty A McLoughlin C Pagnini F Yardley L Dennison L. Experiences of psychological interventions in neurodegenerative diseases: a systematic review and thematic synthesis. *Health Psychol Rev.* (2022): [Online ahead of print]. 10.1080/17437199.2022.2073901 35546326

[19] Morrison L Muller I Yardley L Bradbury K. The person-based approach to planning, optimising, evaluating and implementing behavioural health interventions. *Eur Health Psychol.* (2018) 20:464–9.

[20] Yardley L Ainsworth B Arden-Close E Muller I. The person-based approach to enhancing the acceptability and feasibility of interventions. *Pilot Feasibility Stud.* (2015) 1:37. 10.1186/s40814-015-0033-z 27965815 PMC5153673

[21] Yardley L Morrison L Bradbury K Muller I. The person-based approach to intervention development: application to digital health-related behavior change interventions. *J Med Internet Res.* (2015) 17:e30. 10.2196/jmir.4055 25639757 PMC4327440

[22] Folkman S Greer S. Promoting psychological well-being in the face of serious illness: when theory, research and practice inform each other. *Psychooncology.* (2000) 9:11–9. 10.1002/(SICI)1099-1611(200001/02)9:1<11::AID-PON424>3.0.CO;2-Z 10668055

[23] Díaz J Sancho J Barreto P Bañuls P Renovell M Servera E. Effect of a short-term psychological intervention on the anxiety and depression of amyotrophic lateral sclerosis patients. *J Health Psychol.* (2016) 21:1426–35. 10.1177/1359105314554819 25370571

[24] Pagnini F Marconi A Tagliaferri A Manzoni G Gatto R Fabiani V Meditation training for people with amyotrophic lateral sclerosis: a randomized clinical trial. *Eur J Neurol.* (2017) 24:578–86. 10.1111/ene.13246 28229508

[25] Pinto C Geraghty A Yardley L Dennison L. Emotional distress and well-being among people with motor neurone disease (MND) and their family caregivers: a qualitative interview study. *BMJ Open.* (2021) 11:e044724. 10.1136/bmjopen-2020-044724 34404695 PMC8372816

[26] Geraghty A Muñoz R Yardley L Mc Sharry J Little P Moore M. Developing an unguided internet-delivered intervention for emotional distress in primary care patients: applying common factor and person-based approaches. *JMIR Ment Health.* (2016) 3:e5845. 10.2196/mental.5845 27998878 PMC5209611

[27] Jenkinson C Fitzpatrick R Brennan C Swash M. Evidence for the validity and reliability of the ALS assessment questionnaire: the ALSAQ-40. *Amyotroph Lateral Scler Other Motor Neuron Disord.* (2000) 1:33–40. 10.1080/146608299300080022 12365067

[28] Higginson I Gao W Jackson D Murray J Harding R. Short-form Zarit caregiver burden interviews were valid in advanced conditions. *J Clin Epidemiol.* (2010) 63:535–42. 10.1016/j.jclinepi.2009.06.014 19836205

[29] Braun V Clarke V. Using thematic analysis in psychology. *Qual Res Psychol.* (2006) 3:77–101. 10.1191/1478088706qp063oa 32100154

[30] Bilenchi V Banfi P Pagnini F Volpato E. Psychoeducational groups for people with amyotrophic lateral sclerosis and their caregiver: a qualitative study. *Neurol Sci.* (2022) 43:4239–55. 10.1007/s10072-022-05930-2 35156152

[31] Foley G Timonen V Hardiman O. Understanding psycho-social processes underpinning engagement with services in motor neurone disease: a qualitative study. *Palliat Med.* (2014) 28:318–25. 10.1177/0269216313512013 24637571

[32] Foley G Timonen V Hardiman O. Exerting control and adapting to loss in amyotrophic lateral sclerosis. *Soc Sci Med.* (2014) 101:113–9. 10.1016/j.socscimed.2013.11.003 24560231

[33] Ando H Ashcroft-Kelso H Halhead R Chakrabarti B Young C Cousins R Experience of telehealth in people with motor neurone disease using noninvasive ventilation. *Disabil Rehabil Assist Technol.* (2021) 16:490–6. 10.1080/17483107.2019.1659864 31512539

[34] Weisser F Bristowe K Jackson D. Experiences of burden, needs, rewards and resilience in family caregivers of people living with motor neurone disease/amyotrophic lateral sclerosis: a secondary thematic analysis of qualitative interviews. *Palliat Med.* (2015) 29:737–45. 10.1177/0269216315575851 25762578

[35] Stroebe M Schut H. The dual process model of coping with bereavement: rationale and description. *Death Stud.* (1999) 23:197–224. 10.1080/074811899201046 10848151

[36] Hulbert-Williams N Storey L Wilson K. Psychological interventions for patients with cancer: psychological flexibility and the potential utility of acceptance and commitment therapy. *Eur J Cancer Care.* (2015) 24:15–27. 10.1111/ecc.12223 25100576

[37] Borghouts J Eikey E Mark G De Leon C Schueller S Schneider M Barriers to and facilitators of user engagement with digital mental health interventions: systematic review. *J Med Internet Res.* (2021) 23:e24387. 10.2196/24387 33759801 PMC8074985

[38] Bentley B O’Connor M Williams A Breen L. Dignity therapy online: piloting an online psychosocial intervention for people with terminal illness. *Digit Health.* (2020) 6:2055207620958527. 10.1177/2055207620958527 33014409 PMC7509717

