# Supplementary material for: How do people with MND and caregivers experience a digital mental health intervention? A qualitative study
Source: Front Psychiatry. 2023 Feb 2;14:1083196. doi: 10.3389/fpsyt.2023.1083196 (PMC9932191; doi:10.3389/fpsyt.2023.1083196)
Supplement: Supplementary file 1 [file Table_1.DOCX]

Supplementary material 1: Intervention planning table

| Key issue/ barrier/facilitator to target behaviour (source) | Intervention component or section | Suggested intervention ingredients | Agreed intervention ingredients | Rationale (source) |
| --- | --- | --- | --- | --- |
| Intervention Content | | | | |
| Patients and caregivers feel that hope and positivity helps them cope with things emotionally (1)  Barrier: People might find positivity difficult because of increasing symptom burden but stressed that being positive was still important for coping and wellbeing (1) | Section on building positivity and meaning  Aim: To help users frame negative situations more positively, highlight positive aspects of experiences and help users create or emphasize meaningful aspects of life | - Recognizing positive things, using positive reframing to identify the positive aspects of a negative situation. - Give examples from other people with MND. Acknowledge the difficulty in doing this or reality of situation but say how positive reframing can help - Build hope and meaning by talking about the importance of values and goals, meaningful or value based goals | Building positivity section  Activities in this section: Pleasant activities, Benefit finding, Values and Goals | Some evidence for the effectiveness of positive savouring or benefit finding on psychological outcomes in neurological conditions (2) and in other chronic illness found to be more useful for dealing with boosting positive affect than dealing with negative affect (3).  Focusing on meaning and values is important with palliative care patients (4) and emphasizing meaning and goals along with positive reappraisal has been useful with older adults (5). |
| Patients and caregivers find it difficult dealing with distressing thoughts about the past and future and associated negative feelings (e.g. worry, sadness) (1, 6) | Section on coping with difficult thoughts and feelings  Aim: To equip users with techniques and tips to manage distressing thoughts about the past and future and associated feelings. | - Could divide into emotions (worry, depression/sadness, anger, fear, hopelessness/disappointment, frustration) and relevant strategies - Information about each emotion with relevant MND examples   Examples of strategies are:   - Encourage focus on the present (Mindfulness) - Strategies for dealing with thinking traps (e.g. predicting future, rumination) - Relaxation, distraction | Two sections on dealing with difficult thoughts and emotions:   1. Adjusting to changes – information and strategies to deal with anger, sadness and frustration 2. Dealing with worry and stress   Emotion regulation strategies: Thought distancing (ACT), mindfulness and relaxation exercises | Some promising evidence of the effectiveness of mindfulness in improving psychological outcomes with neurological diseases (2, 7) and some evidence of the effectiveness of ACT (8).  CBT using goal setting and relaxation/visualisation was effective for managing anxiety in advanced cancer (9). |
| Patients and caregivers express feeling tired/overwhelmed with MND and MND care, difficulty coping with the constant changes/losses (1) | Section on dealing with stress or coping with many changes and feelings of burden  Aim: To give people tools/tips to deal with stress and feelings of being burdened or overwhelmed | - Relaxation exercises (providing an escape from current situation) - Pacing self and activities - Practical suggestions for dealing with stress (e.g. taking a break, distracting self, exercising) - Encouraging self-compassion/kindness | Cover feeling overwhelmed in the ‘dealing with worry and stress’ section. Mindfulness exercises (e.g. safe place meditation) and strategies from compassion-focused therapy (compassion break, befriending yourself, self-kindness letter) | Mindfulness and relaxation has some evidence of effectiveness (see studies in previous row)  Self compassion is helpful in improving adaptive coping (10, 11) and also psychological outcomes in cancer patients (12). |

1. Pinto C, Geraghty AW, Yardley L, Dennison L. Emotional distress and well-being among people with motor neurone disease (MND) and their family caregivers: a qualitative interview study. BMJ open. 2021;11(8):e044724.

2. Lai S-T, Lim K-S, Low W-Y, Tang V. Positive psychological interventions for neurological disorders: a systematic review. The Clinical Neuropsychologist. 2019;33(3):490-518.

3. De Ridder D, Geenen R, Kuijer R, van Middendorp H. Psychological adjustment to chronic disease. The Lancet. 2008;372(9634):246-55.

4. Breitbart W, Rosenfeld B, Pessin H, Applebaum A, Kulikowski J, Lichtenthal WG. Meaning-centered group psychotherapy: an effective intervention for improving psychological well-being in patients with advanced cancer. Journal of clinical oncology. 2015;33(7):749.

5. Nowlan JS, Wuthrich VM, Rapee RM. Positive reappraisal in older adults: a systematic literature review. Aging & Mental Health. 2015;19(6):475-84.

6. Pagnini F. Psychological wellbeing and quality of life in amyotrophic lateral sclerosis: a review. International Journal of Psychology. 2013;48(3):194-205.

7. Pagnini F, Marconi A, Tagliaferri A, Manzoni GM, Gatto R, Fabiani V, et al. Meditation training for people with amyotrophic lateral sclerosis: a randomized clinical trial. European journal of neurology. 2017;24(4):578-86.

8. Hulbert‐Williams NJ, Storey L, Wilson KG. Psychological interventions for patients with cancer: psychological flexibility and the potential utility of Acceptance and Commitment Therapy. European journal of cancer care. 2015;24(1):15-27.

9. Uitterhoeve R, Vernooy M, Litjens M, Potting K, Bensing J, De Mulder P, et al. Psychosocial interventions for patients with advanced cancer–a systematic review of the literature. British Journal of Cancer. 2004;91(6):1050-62.

10. Sirois FM, Molnar DS, Hirsch JK. Self-compassion, stress, and coping in the context of chronic illness. Self and Identity. 2015;14(3):334-47.

11. Terry ML, Leary MR. Self-compassion, self-regulation, and health. Self and identity. 2011;10(3):352-62.

12. Pinto‐Gouveia J, Duarte C, Matos M, Fráguas S. The protective role of self‐compassion in relation to psychopathology symptoms and quality of life in chronic and in cancer patients. Clinical psychology & psychotherapy. 2014;21(4):311-23.
