# Supplementary material for: How do people with MND and caregivers experience a digital mental health intervention? A qualitative study
Source: Front Psychiatry. 2023 Feb 2;14:1083196. doi: 10.3389/fpsyt.2023.1083196 (PMC9932191; doi:10.3389/fpsyt.2023.1083196)
Supplement: Supplementary file 2 [file Table_2.DOCX]

Supplementary material 2: Intervention guiding principles

| Key issue/barrier | Design objective | Key features of intervention |
| --- | --- | --- |
| Intervention Tone and Presentation | | |
| People with MND have different levels of disability, particularly with using their hands and voice, difficulties with cognition, and symptoms and ability deteriorate rapidly | To make the intervention easy to use and navigate for people with MND with varying levels of disability | - Make sure suggested techniques or tips take into account varying levels of disability and offer alternative suggestions. - Reassure people that they can modify tasks/suggestions and that there is no best way of doing things - Navigation should be simple and easy to understand, and activities/exercises should not involve too much typing (only clicking) |
| Difficulty finding hope and positivity because of no cure and progressive nature of disease (1) | To make the intervention tone positive and empowering | - Emphasis on what CAN be done to deal with difficult thoughts and emotions - Make sure language used is optimistic but also realistic - Use messages of positive reinforcement at different stages - Not to sound confronting about death/future symptoms |
| People talk about the usefulness of being understood and empathy, from professionals and friends and family (1) | To demonstrate empathy with people with MND and the difficulties associated with coping with MND | - When making suggestions of techniques or activities, acknowledge difficulties/effort with doing them - Make sure examples and information are MND specific - Provide quotes from other people with MND and caregivers to accompany information and strategies |
| People are already burdened with the effort of living with MND and MND care (1, 2) | The intervention should not be an additional burden for people with MND and carers | - Intervention should not have too much information or reading to reduce cognitive effort - Intervention should not seem like a chore – no prescribed way of using it, no fixed sessions or tasks. If there are exercises or tasks to try out on their own, these should be simple to do and there should be an option not to do them for people who don’t want to - Intervention should not involve the mastery of difficult skills, instead information and activities should be short and easy to do |
| People may have different psychological issues that they are dealing with at different stages (e.g. at diagnosis or later coping with new losses). The way people cope/preference for coping with these issues are also highly individual. (1, 2) | To be flexible to use at different points of the disease, for different psychological issues and for people with different preferences for coping | - Allow people to choose content based on what is most relevant to them - Emphasize that people cope differently and there is no ‘right’ way - Give people options of different techniques/different practical ways to handle the situation/emotion - Cover a variety of issues that people go through at different stages - Provide links to additional psychological support in case people need this |

1. Pinto C, Geraghty AW, Yardley L, Dennison L. Emotional distress and well-being among people with motor neurone disease (MND) and their family caregivers: a qualitative interview study. BMJ open. 2021;11(8):e044724.
2. Pinto C, Geraghty AW, McLoughlin C, Pagnini F, Yardley L, Dennison L. Experiences of psychological interventions in neurodegenerative diseases: a systematic review and thematic synthesis. Health Psychology Review. 2022 May 12:1-23.
