# Supplementary material for: How do people with MND and caregivers experience a digital mental health intervention? A qualitative study
Source: Front Psychiatry. 2023 Feb 2;14:1083196. doi: 10.3389/fpsyt.2023.1083196 (PMC9932191; doi:10.3389/fpsyt.2023.1083196)
Supplement: Supplementary file 3 [file Table_3.DOCX]

Supplementary material 3: Think-aloud interview topic guide

**Questions about the website introduction**

- What do you think about the title of the website?
- (On each page of this section) What are your thoughts about this page?
  - Is the information clear?
  - Was there anything you didn’t understand?
- (At the end of the introduction section) What did you think about the introduction?
  - Was anything unclear?
  - Is there anything else you would’ve liked to know at this stage?

**Questions for each page of the website**

(ask 2-3 questions from the list based on the particular page and the participant’s navigation/response)

- What are your first impressions of this page?
- What are you thinking now?
- What option would you choose?
- What made you choose that option?
- What do you think about this information/activity/idea?
- Is there anything you like/don’t like about this page? What is it about that, that you like/don’t like?
- I noticed you (smiled/frowned/hesitated). Can you tell me what you thought about that?

**Questions at the end, after going through the website**

- Overall, what did you think about the website?
- Can you tell me anything you particularly liked about the website?
- Can you tell me about anything you disliked or were less keen on?
- Having gone through the website, can you tell me how you feel about using this website?
- Are there any things we need to keep in mind/pay attention when we are designing websites like this for people with MND or family members/caregivers?
- Is there anything else you would like to mention about the website?
