# Supplementary material for: How do people with MND and caregivers experience a digital mental health intervention? A qualitative study
Source: Front Psychiatry. 2023 Feb 2;14:1083196. doi: 10.3389/fpsyt.2023.1083196 (PMC9932191; doi:10.3389/fpsyt.2023.1083196)
Supplement: Supplementary file 4 [file Table_4.DOCX]

Supplementary material 4: Extract from table of changes

| Location in intervention | Negative comments/ suggested changes | Positive comments | Possible change | Reason for change | Agreed change | MoScow (Must, Should, Could, Would) |
| --- | --- | --- | --- | --- | --- | --- |
| Homepage | Signpost people to additional activities, make this as an introduction to mental wellbeing for people with MND and then they can try other activities to deepen their understanding. Because people may just skim the website and you don’t want people don’t feel this is all the support or all the techniques available (F4) |  | Potentially can add a section on the homepage if people need additional support. This could include both online resources or how to access face-to-face psychological support | EAS | Added a section directing people to resources for professional and peer support | Could |
| Navigation | It would be good to have a link to the ‘All techniques’ there, rather than going to the main menu (F3, 53:22) Minimizing the amount of clicks is important if you are using an eye gaze system, so it’s less frustrating, especially if they are asking someone to help them (P7, 54:00) | The rest of your website is good for eye gaze because the font size is big and easy to read, not overcrowded and the buttons are big to click (P7, 55:27) | Wherever possible, if I have mentioned something from another section, add a link to it | IMP – in line with guiding principles | Hyperlinks added throughout website to make navigation easier | Must |
| Section on Anger | It sounds patient centric rather than carer centric (F7, p2 6:35) |  | Change wording to focus on patient and carer experience | IMP, REP | Changed tense to ‘you’ to make it relevant to both patients and caregivers.  Added caregiver quote on this page as well | Must |
| Practical tips | Physical activity may be difficult for some, instead saying ‘going out in nature’ Going into a park or looking at a tree, nature takes away anger (F4) | Useful to have something to take away and apply in your life (F2) | Say ‘going out into nature for exercise or to take a break’ | IMP (in line with our guiding principles) | Rephrased to ‘getting some fresh air’ and ‘spending time with nature’ | Must |
| Stress and feeling burdened section | Feeling burdened is not clear, it’s quite a negative word. Feeling overwhelmed might be better (F6, 22:13) |  | Change title to ‘feeling overwhelmed’ | EAS, NCON | ‘Stress and feeling overwhelmed’ | Should |
| Pleasant activities | It may be helpful to say ‘when you are planning your list, it may be helpful to discuss with your friend or family member’ so they can help with any preparations or adaptations (F3, 1:24:28) |  | Added a phrase saying it might be helpful to include family members if you need any help with these activities | EAS, IMP | Added a phrase saying it might be helpful to include family members if you need any help with these activities | Should |
| Benefit finding | It’s more about positive thinking or positive mindset than benefit finding.  Benefit finding sounds like looking for disability benefits (F6, 39:34) |  | Consider changing name of activity, currently confusing | REP | Name changed to 'Finding positives' | Must |

^*^ IMP – Important change; EAS – easy and uncontroversial; REP – repeated by many participants; NC – Not changed (explain why);

EXP – supported by experience (PPI or experts or literature); NCON – does not contradict evidence, logic model or guiding principles
