# Supplementary material for: How do people with MND and caregivers experience a digital mental health intervention? A qualitative study
Source: Front Psychiatry. 2023 Feb 2;14:1083196. doi: 10.3389/fpsyt.2023.1083196 (PMC9932191; doi:10.3389/fpsyt.2023.1083196)
Supplement: Supplementary file 5 [file Table_5.DOCX]

Supplementary material 5: Study 2 interview topic guide

**Opening question**

- To start with, can you just tell me a bit about how you’ve got on with trying out the CALM website.

**Questions about overall website use**

- Can you tell me about when you used the website?
- Can you tell me a bit more about how often you logged in and what made you decide to log in?
- Can you talk me through the sections/activities you looked at? How did you decide to choose them?
- Were there any sections/activities that you did not look at? Or that you used least in the whole website? Can you tell me a bit more about that?
- Can you tell me about whether you had a go at trying any of the techniques or suggestions from the CALM website? How did that go?
- Could you tell me about any part of the website that didn’t seem to work properly or that you had any problems with?
- (Based on the website usage data) I can see that you often looked at X section, or did not look at X section, or logged in X number of times. Can you tell me a bit more about this?

**Questions about each section** (ask questions after looking at usage metrics, which sections/activities were used, how often)

- **What did you think of** (5 sections - the introduction, building positivity and meaning, adjusting to changes, dealing with worries and stress, all activities)
  1. Can you describe whether this section felt relevant to you?
  2. Can you describe anything you liked about this section?
  3. Can you tell me about anything you disliked about this section?
  4. Was there anything that was useful to you in this section?
  5. Was there anything that was not useful to you in this section?
  6. Can you tell me about anything that you would change? Or anything that didn’t make sense to you?
  7. How did you find the activities or suggested techniques? [name and summarise the specific techniques here]

**Questions about dealing with emotions after using the CALM website**

- After having used the website for some time, how do you now feel about dealing with your emotions?
- Can you tell me about any advice or technique from the website that you think you might use now, or in the future?
- Has anything changed with you since you have been going through the website?
- How has it been for you using this website during all the disruption and limitations we’ve had because of COVID 19?

Is there anything else you would like to mention about the website/advice and activities?
